# The Complete Chloroplast Genome of *Dioscorea hypoglauca*: Comparative Genomics and Phylogenetic Insights

**DOI:** 10.1002/ece3.74420

**Published:** 2026-10-11

**Authors:** Gang Li, Daofeng Gong, Xiang Cheng, Meng Li, Na Chen, Lei Cheng

**Affiliations:** ^1^ Bozhou Vocational and Technical College Bozhou China

**Keywords:** chloroplast genome, comparative genomics, *Dioscorea hypoglauca*, phylogenetic analysis, simple sequence repeats (SSRs)

## Abstract

The complete chloroplast genome of *Dioscorea hypoglauca*—a medicinal plant native to southern China—was assembled and characterized. The genome is circular, 153,219 bp in length, and follows the typical quadripartite organization: a large single‐copy (LSC) region of 83,692 bp, a small single‐copy (SSC) region of 18,813 bp, and two inverted repeat (IR) regions, each 25,357 bp. In total, 129 genes were annotated, including 84 protein‐coding genes, 37 tRNA genes, and eight rRNA genes. Thirty‐five simple sequence repeats (SSRs) were detected; most were mononucleotide A/T repeats. A comparison with 22 other *Dioscorea* species showed that IR boundaries vary considerably across the genus. One notable finding is the boundary shift of the *ycf1* gene in *D. hypoglauca* , which has not been observed in other examined species. Nucleotide diversity (Pi) analysis revealed that intergenic spacers‐especially *rpl22‐rps19* (Pi = 0.11204)‐are highly variable, whereas tRNA genes are extremely conserved. Codon usage analysis indicated a strong bias toward A/T‐ending codons. Phylogenetic reconstruction based on complete plastomes (using maximum likelihood and Bayesian inference) placed *D. hypoglauca* as a close relative of *D. aspersa* and *D. pyrifolia*. Overall, these results clarify the systematic position of *D. hypoglauca* , illustrate the dynamic evolution of IR regions within *Dioscorea*, and provide a genomic resource for future evolutionary and population genetic studies.

## Graphical abstract

We assembled and characterized the complete chloroplast genome of *Dioscorea hypoglauca*, a medicinally important yam species from southern China. The 153,219 bp genome exhibits a typical quadripartite structure, containing 129 genes (84 protein‐coding, 37 tRNA, and 8 rRNA genes), and comparative analyses with 22 other Dioscorea species revealed dynamic IR boundary shifts, including a unique ycf1 boundary shift, along with highly variable intergenic spacers as candidate DNA barcodes. Phylogenetic reconstruction placed *D. hypoglauca* as a close relative of *D. aspersa* and *D. pyrifolia*, clarifying its systematic position within the genus.

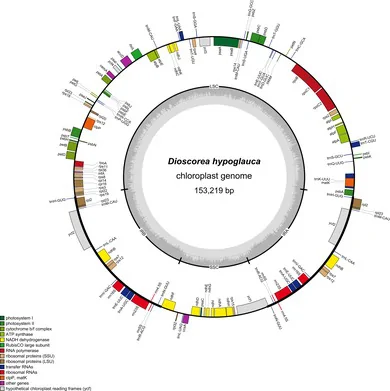

## 1 Introduction

*Dioscorea* L. is the largest genus in the family Dioscoreaceae. It includes about 600 species, distributed across tropical and subtropical regions of Southeast Asia, Africa, and South America (Huber 1998; Caddick et al. 2002). Yams are not only staple starch crops but also medicinal resources (Kumar et al. 2017; Shen et al. 2018).

Among them, *Dioscorea hypoglauca* Palibin (currently treated as a distinct species in the Chinese Pharmacopeia and Chinese flora; accepted as *Dioscorea collettii* var. *hypoglauca* (Palibin) C.T.Ting in some international databases) is a perennial twining herb. Its dried rhizome is officially listed in the Chinese Pharmacopeia as “Fen Bi Xie” (粉萆薢). Traditionally, the rhizome is collected in autumn and winter, cleaned, sliced, and dried. It is used to remove dampness, clear turbidity, expel wind, and relieve arthralgia. Clinical applications include treating turbid urine, leukorrhea, rheumatic pain, joint stiffness, and lumbar and knee soreness (Chinese Pharmacopeia 2025). Modern phytochemical studies show that *D. hypoglauca* contains steroidal saponins, diosgenin, and other bioactive compounds with anti‐inflammatory, and anti‐hyperuricemic activities (Qin et al. 2025).

However, accurate identification and taxonomic delimitation of this species remain difficult. The plants are dioecious; their flowers are inconspicuous, and their morphology varies greatly. These features often cause confusion with other sympatric *Dioscorea* species (Wilkin et al. 2005; Hsu et al. 2013).

The chloroplast (cp) genome in angiosperms is typically 120–170 kb. It has a conserved quadripartite structure: a large single‐copy (LSC) region, a small single‐copy (SSC) region, and two inverted repeat (IR) regions (Shaw et al. 2007; Wicke et al. 2011). Because of its maternal inheritance, moderate evolutionary rate, and structural stability, the cp genome is widely used for phylogenetic reconstruction, authentication of medicinal plants, and population genetics (Daniell et al. 2016, 2021).

With the rapid development of next‐generation sequencing (NGS), complete cp genomes can now be assembled and compared efficiently. This has allowed the identification of highly variable regions (e.g., *trnC‐ndhE*, *ycf1*, *rpl32*) as potential super‐barcodes for species discrimination in complex genera like *Dioscorea* (Zhao et al. 2018; Xia et al. 2019). Comparative genomic analyses have also revealed dynamic evolutionary events, including IR boundary expansion and contraction, which can lead to gene rearrangements, duplications, or losses (Zhu et al. 2016). Previous studies on Dioscoreaceae indicate that, despite general conservation, lineage‐specific variations occur at the LSC/IRb and SSC/IRa junctions, affecting genes such as *rps19*, *ndhF*, and *ycf1* (Zhao et al. 2018).

To date, complete cp genomes have been reported for many *Dioscorea* species (Zhao et al. 2018; Wonok et al. 2023), but none is available for *D. hypoglauca* . This gap limits accurate molecular identification, phylogenetic placement, and evolutionary understanding of this medicinal species. Here we sequenced, assembled, and annotated the complete cp genome of *D. hypoglauca* using the Illumina HiSeq platform. We analyzed its genome structure, GC content, gene composition, SSR loci, and codon usage bias, and compared it with 22 closely related *Dioscorea* species. We also reconstructed the phylogeny of 33 Dioscoreaceae taxa based on complete cp genome sequences. These results provide genomic resources for authenticating and conserving *D. hypoglauca* , and they support future work on the evolutionary dynamics and species delimitation of the genus *Dioscorea*.

## 2 Materials and Methods

### 2.1 Plant Materials and DNA Extraction

Leaf samples of *Dioscorea hypoglauca* Palibin were collected from a mountainous area in northern Guangdong Province, China (about 24.8° N, 113.0° E), in October 2024. Species identification followed the morphological criteria described in the Flora of China (Wu and Raven 2000). Voucher specimens were placed in the Herbarium of Bozhou Vocational and Technical College (collection number BZ‐20251201). Once collected, the leaves were quickly dried in silica gel beads and taken to the lab for DNA extraction.

Total genomic DNA was extracted from about 50 mg of dried leaf tissue. We used a magnetic‐bead‐based method with the CretMag Multi Sample DNA Kit (Suzhou Cretaceous Biotechnology Co. Ltd., Suzhou, China), following the manufacturer's instructions (Zhou et al. 2022). DNA purity and concentration were checked with a NanoDrop One spectrophotometer (Thermo Fisher Scientific, Waltham, MA, USA) and a Qubit 4.0 fluorometer (Thermo Fisher Scientific). The extracted DNA had an A260/A280 ratio of 1.82 and a concentration of 85.6 ng/μL. On a 1% agarose gel, it showed clear high‐molecular‐weight bands, with no obvious smearing—so the DNA quality was considered good enough for library preparation.

### 2.2 Sequencing, Genome Assembly and Annotation

A paired‐end library (insert size 350 bp) was prepared using the VAHTS Universal Plus DNA Library Prep Kit (Nanjing Vazyme Biotech Co. Ltd., Nanjing, China). Sequencing was done on an Illumina HiSeq 4000 platform (Shanghai OE Biotech Co. Ltd., Shanghai, China), with 150 bp paired‐end reads. This produced roughly 6.8 Gb of raw data.

Raw reads were first processed with FastQC v0.12.0 (http://www.bioinformatics.babraham.ac.uk/projects/fastqc/) to assess quality. We then trimmed adapter sequences and low‐quality bases (*Q* < 20) using fastp v0.23.2 (Chen et al. 2018).

The complete chloroplast genome of *D. hypoglauca* was assembled with GetOrganelle v1.7.7.1 (https://github.com/Kinggerm/GetOrganelle) (Jin et al. 2020), using the cp genome of *Dioscorea brevipetiolata* (GenBank accession NC_062811.1) as a reference. Default parameters were used, and k‐mer sizes 21, 45, 67, 85, and 105 were tested. Annotation was performed with the CPGAVAS2 online platform (http://www.herbalgenomics.org/cpgavas/) (Shi et al. 2019) and then manually refined using Geneious Prime v2022.0 (Biomatters Ltd., Auckland, New Zealand). The circular genome map was drawn with OGDRAW v1.2 (http://ogdraw.mpimp‐golm.mpg.de/) (Lohse et al. 2007). The complete cp genome sequence of *D. hypoglauca* has been deposited in NCBI under accession number PZ427684.

### 2.3 Identification of Simple Sequence Repeats (SSRs)

SSRs in the *D. hypoglauca* cp genome were detected with MISA‐web (https://webblast.ipk‐gatersleben.de/misa/) (Beier et al. 2017). The detection parameters were: minimum repeats of 10 for mononucleotides, 6 for dinucleotides, 5 for trinucleotides, 5 for tetranucleotides, 5 for pentanucleotides, and 5 for hexanucleotides. These parameters were selected following the MISA manual and common practices in chloroplast genome SSR studies, as the aim was to identify SSR loci for *D. hypoglauca* rather than to perform cross‐species comparison.

### 2.4 Comparative Genome and Nucleotide Diversity Analysis

To investigate structural evolution of the chloroplast genome within the genus *Dioscorea*, we compared the IR boundaries (LSC/IRb, IRb/SSC, SSC/IRa, IRa/LSC) of *D. hypoglauca* with those of 22 other *Dioscorea* species. Their cp genome sequences were retrieved from the NCBI GenBank database, including: *D. banzhuana* (NC_077568.1), *D. biformifolia* (NC_077569.1), *D. brevipetiolata* (NC_062811.1), *D. burkilliana* (NC_039835.1), *D. cirrhosa* (NC_065059.1), *D. collettii* (OQ525992.1), *D. deltoidea* (NC_077564.1), *D. depauperata* (NC_062812.1), *D. elephantipes* (EF380353.1), *D. esculenta* (NC_052854.1), *D. glabra* (NC_062813.1), *D. gracillima* (NC_077565.1), *D. japonica* (MW413805.1), *D. nipponica* (OQ525997.1), *D. oppositifolia* (PX509271.1), *D. polystachya* (PX220302.1), *D. pyrifolia* (NC_062814.1), *D. schimperiana* (NC_039855.1), *D. spongiosa* (NC_077566.1), *D. tenuipes* (PV612491.1), *D. tokoro* (NC_077567.1), and *D. zingiberensis* (NC_027090.1). The cp genome of *Dioscorea polystachya* (PX220302.1) was used as the reference for comparative analysis. Visualization of the IR junctions was performed using IRscope (https://irscope.shinyapps.io/irapp/) (Amiryousefi et al. 2018). Whole‐genome sequence comparisons were done with mVISTA (https://genome.lbl.gov/vista/mvista/submit.shtml) using the Shuffle‐LAGAN mode (Frazer et al. 2004). The reference genome was *D. polystachya*.

Nucleotide diversity (Pi) was calculated from multiple alignments of the complete cp genomes of 23 *Dioscorea* species (including *D. hypoglauca* ). We used DnaSP v6 for this (Rozas et al. 2017). A sliding window analysis was run with a window length of 800 bp and a step size of 200 bp to pick out highly variable regions.

The 23 species for comparative analyses represent all *Dioscorea* species for which complete and consistently annotated plastomes were available in the NCBI database at the time of analysis. The expanded set of 27 species for phylogenetic analysis includes all available *Dioscorea* plastomes in NCBI to improve taxonomic coverage across the genus.

### 2.5 Codon Usage Analysis

Codon usage bias in the protein‐coding sequences (CDS) of *D. hypoglauca* cp genome was analyzed using CodonW v1.4.2 (https://sourceforge.net/projects/codonw/files/codonw/Win32‐Executables‐1.4.2/, accessed: December 25, 2025). Relative synonymous codon usage (RSCU) values were computed following Sharp and Li (1987). According to standard criteria, RSCU = 1.0 indicates no bias, RSCU > 1.0 indicates preferential usage, and RSCU < 1.0 indicates under‐representation.

### 2.6 Phylogenetic Analysis

We reconstructed the phylogeny using 33 complete cp genome sequences. This data set includes the newly sequenced *D. hypoglauca* genome and 32 cp genomes downloaded from NCBI. Three *Aletris* species were set as outgroups: *Aletris megalantha* (NC_054333.1), *A. spicata* (NC_033411.1), and *A. fauriei* (NC_033412.1). The ingroup contains 27 *Dioscorea* species‐the 22 listed in Section 2.4 plus five additional ones from NCBI, full accession numbers are provided in Table S1. The expanded set of 27 species for phylogenetic analysis includes all available *Dioscorea* plastomes in NCBI to improve taxonomic coverage across the genus.

For phylogenetic analysis, we extracted the protein‐coding sequences (CDS) from all 33 plastomes. Only one copy of each duplicated gene in the IR regions was retained. All sequences were aligned with MAFFT v7.505 (Katoh et al. 2019). For maximum likelihood (ML) inference, ModelFinder in IQ‐TREE selected GTR+F+R5 as the best‐fit model under the BIC criterion, and we used this model for tree inference. Bayesian inference (BI) was done with MrBayes v3.2.7a (Ronquist et al. 2012). Two independent runs were conducted, each for 2 million generations, sampling every 100 generations. The first 25% of samples were discarded as burn‐in. The resulting trees were visualized with FigTree v1.4.4 (Rambaut 2014).

## 3 Results

### 3.1 Cp Genome Features of *Dioscorea hypoglauca*

We assembled the complete chloroplast genome of *Dioscorea hypoglauca* from Illumina paired‐end sequencing data. The genome is a circular DNA molecule of 153,219 bp (Figure 1). This length falls within the typical range of previously reported *Dioscorea* cp genomes (152,039–155,406 bp). Like most angiosperms, the *D. hypoglauca* cp genome has a quadripartite structure: a large single‐copy (LSC) region of 83,692 bp, a small single‐copy (SSC) region of 18,813 bp, and two inverted repeat (IR) regions (IRa and IRb) of 25,357 bp each. The overall GC content is 37.0%, which matches other *Dioscorea* species (e.g., *D. brevipetiolata*, 37.0%; *D. glabra* , 37.0%). The IR regions have a GC content of 43.0%, much higher than that of the LSC (35.0%) and SSC (30.5%)—a pattern often seen in cp genomes, probably due to the presence of ribosomal RNA genes with higher GC content.

**FIGURE 1:**
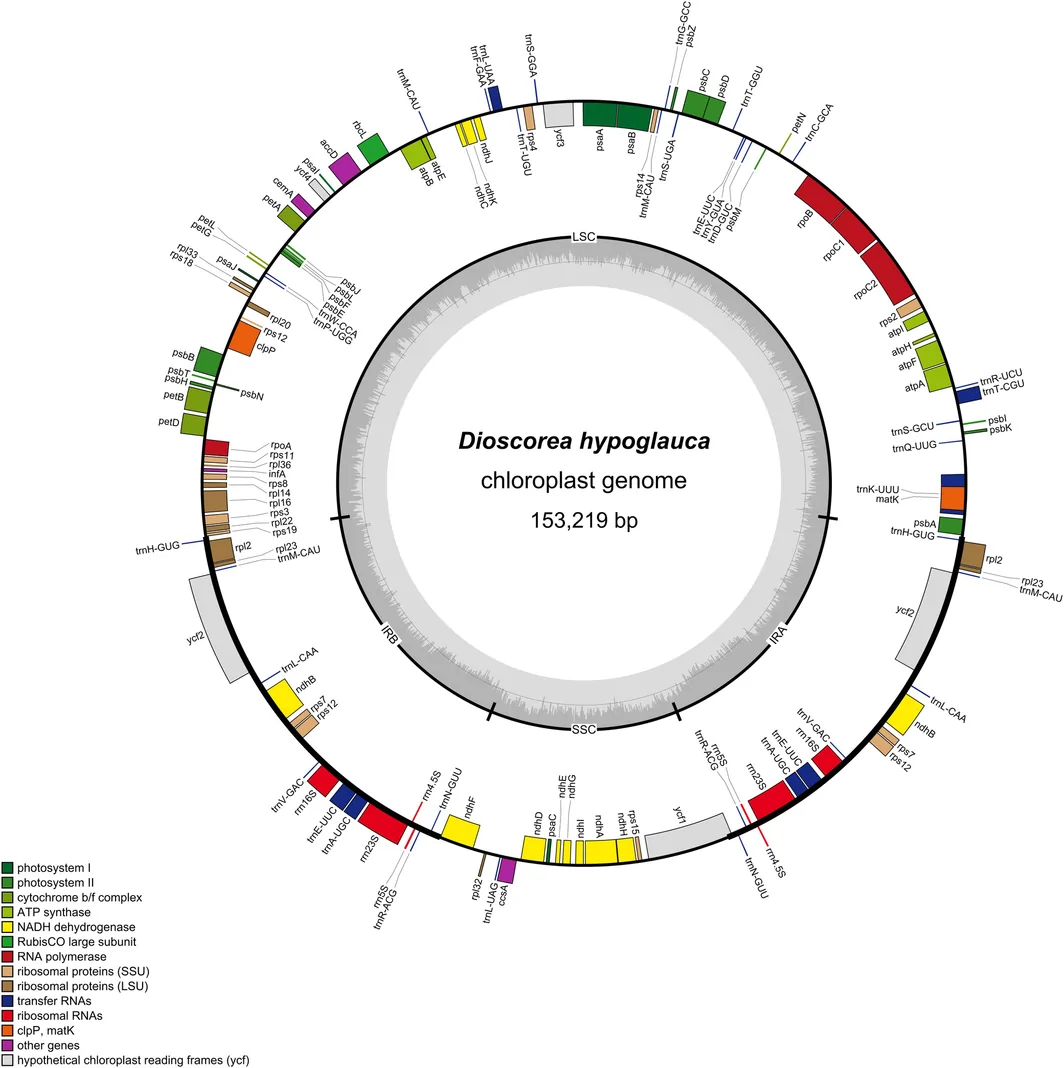
The circular complete cp genome map of *D. hypoglauca* . Genes shown outside the outer circle are transcribed clockwise, whereas those shown inside are transcribed counterclockwise.

We annotated 129 functional genes in the *D. hypoglauca* cp genome. This set includes 84 protein‐coding genes, 37 tRNA genes, and 8 rRNA genes (Table 1, Figure 1). These genes fall into three main functional categories: photosynthesis‐related, self‐replication‐related, and others (Table 1). Among the photosynthesis‐related genes, 15 genes are involved in photosystem II (*psbA*, *psbB*, *psbC*, *psbD*, *psbE*, *psbF*, *psbH*, *psbI*, *psbJ*, *psbK*, *psbL*, *psbM*, *psbN*, *psbT*, *psbZ*), five in photosystem I (*psaA*, *psaB*, *psaC*, *psaI*, *psaJ*), twelve in NADH dehydrogenase (*ndhA*, *ndhB*, *ndhC*, *ndhD*, *ndhE*, *ndhF*, *ndhG*, *ndhH*, *ndhI*, *ndhJ*, *ndhK*), six in the cytochrome b/f complex (*petA*, *petB*, *petD*, *petG*, *petL*, *petN*), six in ATP synthase (*atpA*, *atpB*, *atpE*, *atpF*, *atpH*, *atpI*), and one encoding the large subunit of rubisco (*rbcL*). The self‐replication category includes 11 large ribosomal subunit proteins (*rpl2*, *rpl14*, *rpl16*, *rpl20*, *rpl22*, *rpl23*, *rpl32*, *rpl33*, *rpl36*), 13 small ribosomal subunit proteins (*rps2*, *rps3*, *rps4*, *rps7*, *rps8*, *rps11*, *rps12*, *rps14*, *rps15*, *rps18*, *rps19*), four RNA polymerase subunits (*rpoA*, *rpoB*, *rpoC1*, *rpoC2*), eight rRNA genes (two copies each of *rrn16S*, *rrn23S*, *rrn4.5S*, *rrn5S*), and 37 tRNA genes. The “other genes” group includes *matK*, *clpP*, *cemA*, *accD*, *ccsA*, *infA*, and five conserved open reading frames: *ycf1*, *ycf2*, *ycf3*, and *ycf4*.

**TABLE 1:** The basic cp genomes information of *D. hypoglauca* .

Category	Gene group	Gene name	Number
Photosynthesis	Subunits of photosystem I	*psaB*, *psaA*, *psaI*, *psaJ*, *psaC*	5
	Subunits of photosystem II	*psbA*, *psbK*, *psbI*, *psbM*, *psbD*, *psbC*, *psbZ*, *psbJ*, *psbL*, *psbF*, *psbE*, *psbB*, *psbT*, *psbN*, *psbH*	15
	Subunits of NADH dehydrogenase	*ndhJ*, *ndhK*, *ndhC*, *ndhB*(2)#, *ndhF*, *ndhD*, *ndhE*, *ndhG*, *ndhI*, *ndhA#*, *ndhH*	12
	Subunits of cytochrome b/f complex	*petN*, *petA*, *petL*, *petG*, *petB#*, *petD#*	6
	Large subunit of rubisco	*rbcL*	1
	Subunits of ATP synthase	*atpA*, *atpF#*, *atpH*, *atpI*, *atpE*, *atpB*	6
Self‐replication	Proteins of large ribosomal subunit	*rpl33*, *rpl20*, *rpl36*, *rpl14*, *rpl16#*, *rpl22*, *rpl2*(2)#, *rpl23*(2), *rpl32*	11
	Proteins of small ribosomal subunit	*rps12*(2)##, *rps2*, *rps14*, *rps4*, *rps18*, *rps11*, *rps8*, *rps3*, *rps19*, *rps7*(2), *rps15*	13
	Subunits of RNA polymerase	*rpoC2*, *rpoC1#*, *rpoB*, *rpoA*	4
	Ribosomal RNAs	*rrn16S*(2), *rrn23S*(2), *rrn4.5S*(2), *rrn5S*(2)	8
	Transfer RNAs	*trnK‐UUU*, *trnQ‐UUG*, *trnS‐GCU*, *trnT‐CGU*, *trnR‐UCU*, *trnC‐GCA*, *trnD‐GUC*, *trnY‐GUA*, *trnE‐UUC*(3), *trnT‐GGU*, *trnS‐UGA*, *trnG‐GCC*, *trnM‐CAU*(4), *trnS‐GGA*, *trnT‐UGU*, *trnL‐UAA*, *trnF‐GAA*, *trnW‐CCA*, *trnP‐UGG*, *trnH‐GUG*(2), *trnL‐CAA*(2), *trnV‐GAC*(2), *trnA‐UGC*(2), *trnR‐ACG*(2), *trnN‐GUU*(2), *trnL‐UAG*	37
Other genes	Maturase	*matK*	1
	Protease	*clpP##*	1
	Envelope membrane protein	*cemA*	1
	Acetyl‐CoA carboxylase	*accD*	1
	c‐type cytochrome synthesis gene	*ccsA*	1
	Translation initiation factor	*infA*	1
	Conserved open reading frames	*ycf3*##, *ycf4*, *ycf2*(2), *ycf1*	5
Total			129

*Note:* #: intron number, (*n*): gene copy number.

Because the genome has two IR regions, 18 genes are duplicated. These include *ndhB*, *rpl2*, *rpl23*, *rps7*, *rps12*, *rps19*, *ycf2*, *ycf15*, eight rRNA genes, and several tRNA genes (t*rnA‐UGC*, *trnH‐GUG*, *trnI‐CAU*, *trnI‐GAU*, *trnL‐CAA*, *trnN‐GUU*, *trnR‐ACG*, *trnV‐GAC*). The two copies stay perfectly identical, probably due to copy‐dependent repair through gene conversion between the IRs—a common mechanism in chloroplast genomes.

Intron analysis shows that 19 genes contain introns. Fifteen of them have a single intron: *atpF*, *ndhA*, *ndhB*, *petB*, *petD*, *rpl16*, *rpl2*, *rpoC1*, *rps16*, *trnA‐UGC*, *trnG‐UCC*, *trnI‐GAU*, *trnK‐UUU*, *trnL‐UAA*, and *trnV‐UAC*. Two genes, *clpP* and *ycf3*, each have two introns. The *rps12* gene is trans‐spliced: its 5′ exon is in the LSC region and its 3′ exon lies in the IR region (Figure 1). This arrangement is typical for angiosperms and is consistent with the *rps12* gene structure observed in other *Dioscorea* species and in many angiosperms. The complete cp genome sequence of *D. hypoglauca* has been submitted to the NCBI GenBank database under the accession number: PZ427684.

### 3.2 Analysis of SSRs

We detected 35 simple sequence repeat (SSR) loci in the chloroplast genome of *Dioscorea hypoglauca* using MISA‐web (Beier et al. 2017). These SSRs fall into four types: mononucleotide, dinucleotide, trinucleotide, and complex repeats (Table 2; Figure 2). Mononucleotide repeats are the most abundant—29 out of 35 (82.86%). Dinucleotide, trinucleotide, and complex repeats each appear twice (5.71% each) (Figure 2A). All mononucleotide repeats consist of A or T only; no C or G mononucleotides were found. The A‐type mononucleotides (16 occurrences) were slightly more frequent than T‐type (13 occurrences). The length of mononucleotide repeats ranged from 10 to 14 bp, with the majority (23 SSRs) having a length of 10 or 11 bp (Figure 2B).

**TABLE 2:** Summary of simple sequence repeats (SSRs) identified in the *Dioscorea hypoglauca* chloroplast genome.

SSR type	Repeat motif	Number of SSRs	Proportion (%)	Length range (bp)
Mononucleotide	A	16	45.71	10–14
Mononucleotide	T	13	37.14	10–14
Dinucleotide	TA/AT	2	5.71	12
Trinucleotide	ATA/TAT	2	5.71	15
Complex	—	2	5.71	23–82
Total	—	35	100	—

**FIGURE 2:**
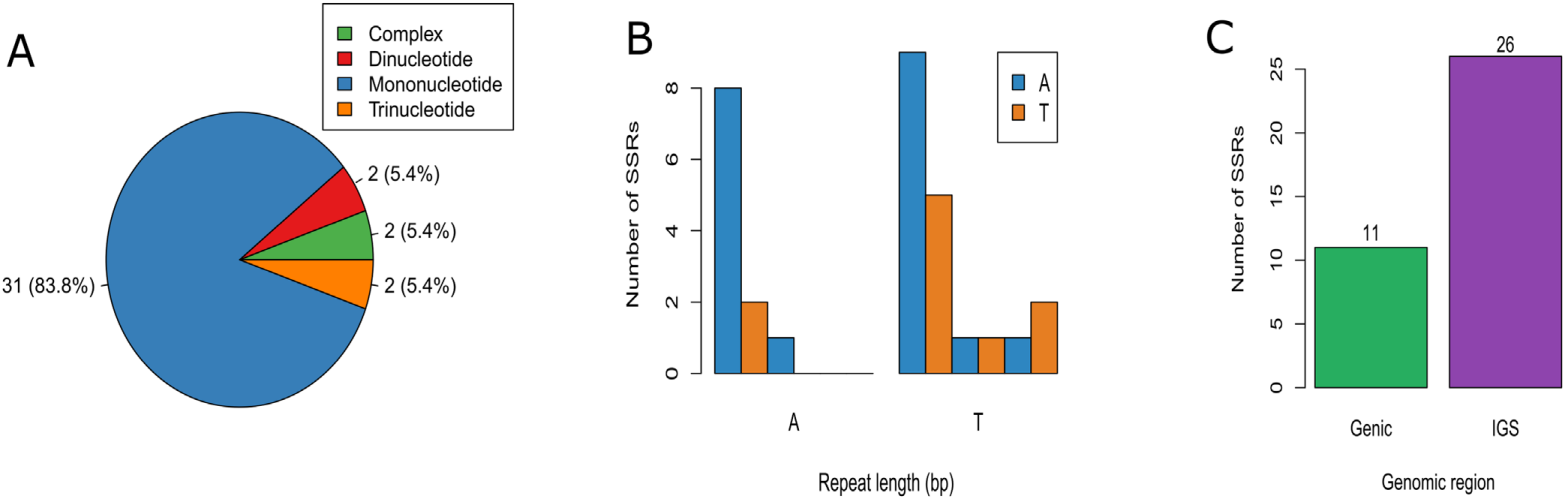
Characterization of SSRs in the *Dioscorea hypoglauca* chloroplast genome. (A) Proportions of different SSR types. (B) Length distribution of mononucleotide SSRs. (C) Distribution of SSRs across genic and intergenic regions.

Two dinucleotide repeats were identified, both of length 6 repeat units (12 bp): one was (TA)6 and the other (AT)6. Two trinucleotide repeats were found, each with 5 repeat units (15 bp): (ATA)5 and (TAT)5. Additionally, two complex repeats were detected: one consisting of (T)10‐ccg‐(A)10 and another longer complex repeat containing (T)11 followed by a 54‐bp insertion and ending with (A)13.

Regarding genomic distribution, most SSRs (26 out of 35) were located in intergenic spacer (IGS) regions, while the remaining nine were situated within genic regions (including introns and coding sequences) (Table 2). Detailed information on each SSR, including its position, repeat motif, length, and flanking region, is provided in Table S2.

### 3.3 Comparative Analysis of Genome Structure in *D. hypoglauca* and Its Related Species

We compared the IR boundaries (LSC/IRb, IRb/SSC, SSC/IRa, and IRa/LSC) of *D. hypoglauca* with those of 22 other *Dioscorea* species to investigate the evolutionary patterns of the chloroplast genome in this genus (Figure 3). All examined cp genomes exhibit the typical quadripartite structure, which suggests that the overall framework is well conserved. However, detailed inspection of the junction regions reveals some lineage‐specific changes in IR expansion or contraction and in gene distribution.

**FIGURE 3:**
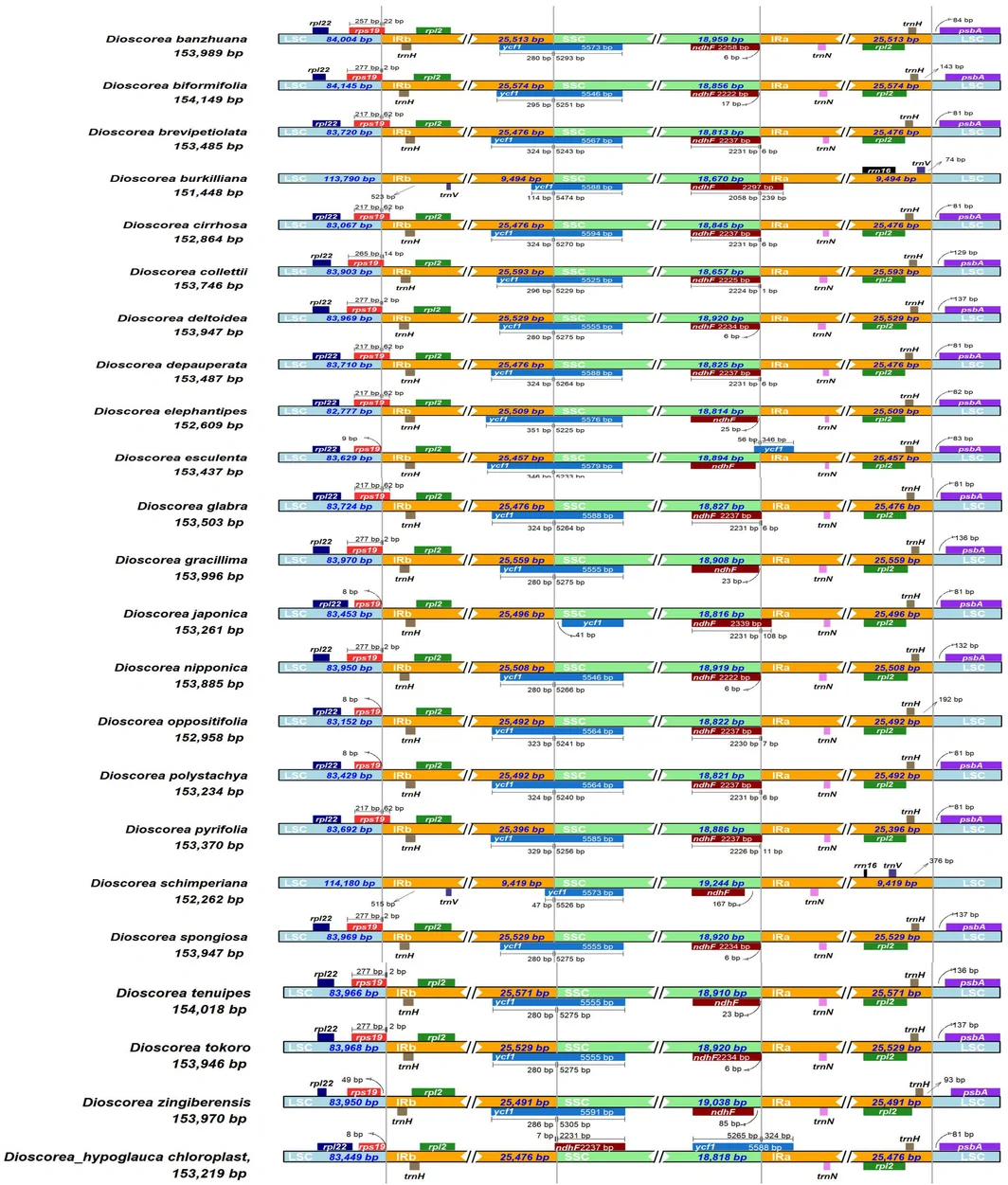
Comparison of the LSC, SSC, and IR boundaries among 23 *Dioscorea* cp genomes. Genes adjacent to the four junctions (JLB, JSB, JSA, JLA) are indicated by colored boxes. The distances between the genes and the boundaries are shown as base pair lengths. The unique boundary shift of *ycf1* in *D. hypoglauca* is highlighted with an asterisk. Boundaries for *D. burkilliana* and *D. schimperiana* are based on NCBI original annotations (accession numbers NC_039835.1 and NC_039855.1).

In most species, *rpl22* is located entirely within the LSC, while *rps19* crosses the LSC/IRb boundary, with a short part extending into IRb. *D. zingiberensis* is notable for its IRb expansion, and *rps19* moved almost completely into IRb (only 49 bp left in LSC). In contrast, *D. hypoglauca* has a relatively contracted IRb, with only 8 bp of *rps19* inside IRb (Figure 3). Thus, the IRb boundary in *D. hypoglauca* appears closer to the ancestral condition, while *D. zingiberensis* has undergone its own expansion.

The *ycf1* gene typically spans the IRb/SSC (JSB) boundary, and most of its sequence lies in the SSC. For instance, in *D. glabra* , the SSC part of *ycf1* is 5588 bp. *D. hypoglauca* is different: its main *ycf1* coding sequence shifted to the IRa region (also 5588 bp), leaving only a short piece in SSC (Figure 3). The average read depth across the *ycf1* boundary region was approximately 245×, consistent with the genome‐wide average coverage. This boundary shift was not observed in any other *Dioscorea* species examined; it may represent a unique structural rearrangement for *D. hypoglauca* .

The *ndhF* gene varies considerably among species. In most of them (e.g., *D. gracillima* ), *ndhF* is completely inside the IRa (2231 bp). In *D. hypoglauca* , it stays inside IRa (2237 bp). But in *D. esculenta* , *ndhF* moved entirely into the SSC—a complete loss of the IRa copy. This indicates that the SSC/IRa boundary is highly dynamic, and the positional shift of *ndhF* may reflect lineage‐specific evolutionary dynamics, but its functional significance remains unclear.

At the IRa/LSC junction (JLA), the positions of *trnH* and *psbA* are useful markers. In most species, *trnH* is in the LSC and *psbA* sits near the boundary. *D. hypoglauca* has the same pattern, with 81 bp between *trnH* and *psbA*. However, *D. schimperiana* has an extra insertion of *rrn16* and *trnV* near JLA, which results in a markedly different gene composition likely a local gain or rearrangement unique to that species.

mVISTA was also used (Figure 4) to compare whole‐genome sequences, with *D. polystachya* as the reference. The results are consistent with the IR boundary analysis. The LSC and SSC regions are generally more divergent than the IRs, which is consistent with the higher evolutionary constraint of IRs due to gene conversion and replication. Several intergenic spacers (*trnK‐trnQ*, *trnS‐trnG*, *trnC‐petN*, *trnE‐trnT*, *trnL‐rpl32*) were identified as hotspots of divergence, especially in LSC and SSC (Figure 4). The *ycf1* and *ndhF* regions also show clear divergence among species, matching the boundary shifts we saw. The mVISTA analysis also shows that *D. hypoglauca* shares a high degree of synteny with other *Dioscorea* species, but it has distinct features at the JSB boundary (where *ycf1* is relocated) and at some intergenic spacers.

**FIGURE 4:**
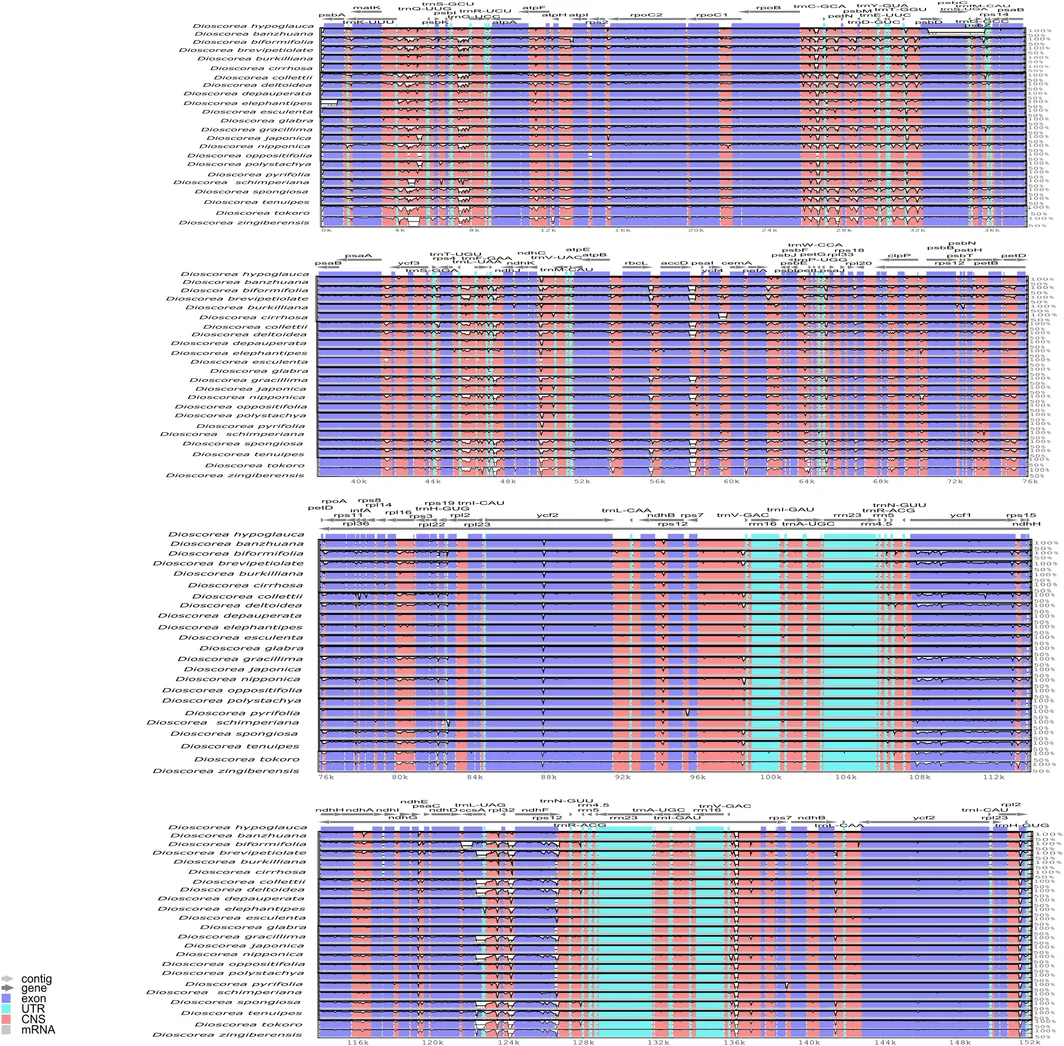
The mVISTA alignment of 23 *Dioscorea* cp genomes using *D. polystachya* as the reference. The x‐axis represents the genomic coordinates, and the y‐axis shows the percentage identity. Gray arrows indicate the direction of transcription of annotated genes. Highly variable intergenic regions are marked with arrows.

In summary, comparing the IR boundaries of *D. hypoglauca* with 22 related species confirms that the genus is structurally conserved, but also uncovers lineage‐specific changes—for example, the unique boundary shift of *ycf1* and the variable positioning of *ndhF*. These variations could serve as molecular markers for species identification and phylogenetic studies. These findings also highlight the dynamic nature the IR boundaries can be in *Dioscorea* and provide a foundation for future studies on adaptation and population genetics of this medicinal genus.

### 3.4 Codon Usage Analysis of *Dioscorea hypoglauca* Cp Genome

We analyzed codon usage bias in the *Dioscorea hypoglauca* chloroplast genome by calculating RSCU (relative synonymous codon usage) for all protein‐coding sequences (CDS). A total of 61 sense codons were found, which code for 20 amino acids (Table 3; Figure 5). The RSCU values are presented in Table 3 and visualized in Figure 5.

**TABLE 3:** Codon usage and codon‐anticodon recognition patterns of *Dioscorea hypoglauca*.

Amino acid	Symbol	Codon	RSCU	Numbers
A	Ala	GCU	1.83	500
A	Ala	GCC	0.59	161
A	Ala	GCA	1.23	336
A	Ala	GCG	0.34	94
C	Cys	UGU	1.54	180
C	Cys	UGC	0.46	54
D	Asp	GAU	1.63	688
D	Asp	GAC	0.37	154
E	Glu	GAA	1.53	870
E	Glu	GAG	0.47	265
F	Phe	UUU	1.26	726
F	Phe	UUC	0.74	423
G	Gly	GGU	1.37	474
G	Gly	GGC	0.38	133
G	Gly	GGA	1.6	555
G	Gly	GGG	0.64	222
H	His	CAU	1.56	381
H	His	CAC	0.44	109
I	Ile	AUU	1.46	886
I	Ile	AUC	0.57	346
I	Ile	AUA	0.97	587
K	Lys	AAA	1.52	812
K	Lys	AAG	0.48	253
L	Leu	UUA	1.88	663
L	Leu	UUG	1.32	466
L	Leu	CUU	1.25	440
L	Leu	CUC	0.4	140
L	Leu	CUA	0.78	275
L	Leu	CUG	0.37	129
M	Met	AUG	1	482
N	Asn	AAU	1.59	803
N	Asn	AAC	0.41	206
P	Pro	CCU	1.53	309
P	Pro	CCC	0.92	186
P	Pro	CCA	1.11	225
P	Pro	CCG	0.44	90
Q	Gln	CAA	1.52	584
Q	Gln	CAG	0.48	185
R	Arg	CGU	1.47	303
R	Arg	CGC	0.33	67
R	Arg	CGA	1.33	274
R	Arg	CGG	0.35	72
R	Arg	AGA	1.9	390
R	Arg	AGG	0.62	127
S	Ser	UCU	1.67	433
S	Ser	UCC	0.98	255
S	Ser	UCA	1.3	339
S	Ser	UCG	0.5	131
S	Ser	AGU	1.27	329
S	Ser	AGC	0.28	72
T	Thr	ACU	1.61	423
T	Thr	ACC	0.72	188
T	Thr	ACA	1.25	328
T	Thr	ACG	0.43	112
V	Val	GUU	1.48	417
V	Val	GUC	0.38	108
V	Val	GUA	1.53	433
V	Val	GUG	0.61	171
W	Trp	UGG	1	375
Y	Tyr	UAU	1.61	643
Y	Tyr	UAC	0.39	157
	Ter	UAA	1.35	23
	Ter	UAG	0.82	14
	Ter	UGA	0.82	14

**FIGURE 5:**
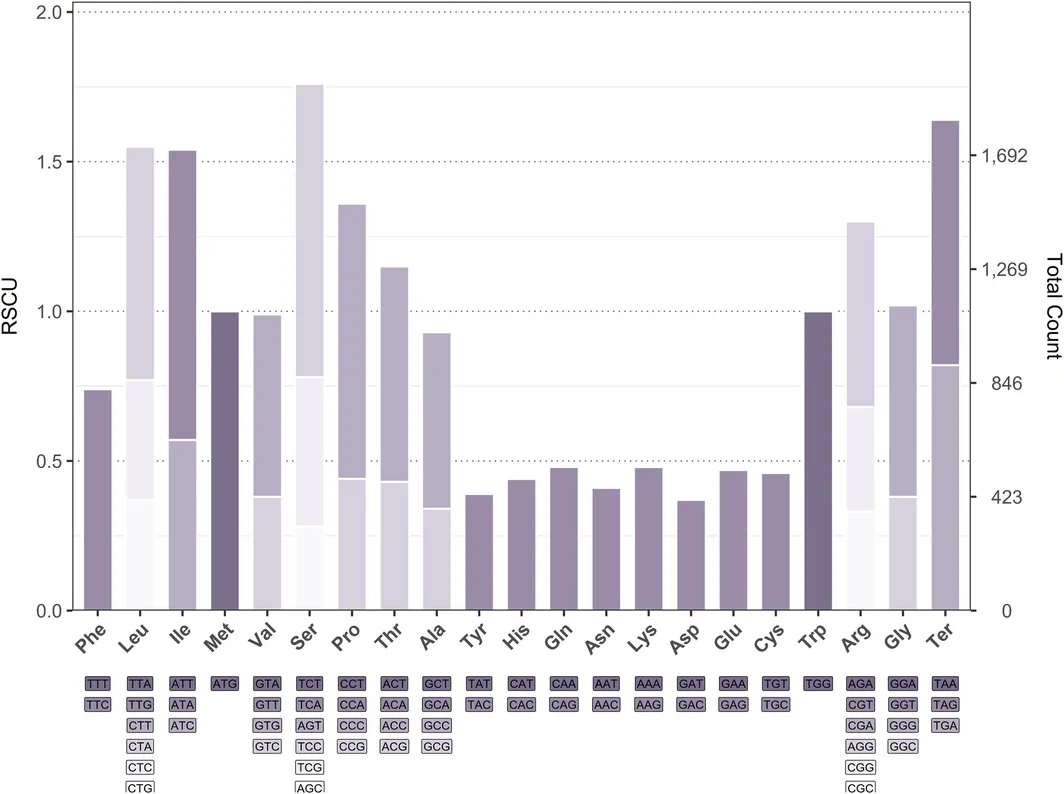
RSCU in the *Dioscorea hypoglauca* chloroplast genome. The left column shows the RSCU value for each codon, and the right column indicates the percentage of total codons for each amino acid. Codons with RSCU > 1.0 are preferentially used, and those with RSCU < 1.0 are under‐represented.

Among the 61 sense codons, 28 have RSCU > 1.0; these are used more frequently. The remaining 33 have RSCU < 1.0, meaning they are used less often. Codons with RSCU = 1.0 were not observed for any sense codon, suggesting a pervasive codon usage bias throughout the plastome. The most frequently used amino acids were leucine (Leu), serine (Ser), and arginine (Arg), whereas methionine (Met) and tryptophan (Trp) showed the lowest frequencies, which is consistent with their single‐codon nature and obligatory RSCU = 1.0 (Table 3).

A strong preference for A/T‐ending codons was evident. For example, among the six codons for leucine, the A/T‐ending codons TTA (RSCU = 1.88) and TTG (RSCU = 1.32) were highly favored, whereas the G/C‐ending codons CTC (0.40) and CTG (0.37) were strongly avoided. For valine, the A/T‐ending codons GTA (1.53) and GTT (1.48) are used more than GTC (0.38) and GTG (0.61). For serine, TCT (1.67), TCA (1.30), and AGT (1.27)—all ending with A or T—appear more frequently; the G/C‐ending codon AGC (0.28) is rare. For arginine, the A/T‐ending codon AGA (1.90) has the highest RSCU value among all codons; CGC (0.33) and CGG (0.35) are the least used. Isoleucine prefers ATT (1.46) over ATC (0.57). Asparagine prefers AAT (1.59) over AAC (0.41). Overall, there is a clear bias toward A/T‐rich codons. This matches the low GC content (37.0%) of the *D. hypoglauca* cp genome.

Among the three stop codons, UAA (RSCU = 1.35) was the most frequently used, followed by UAG (0.82) and UGA (0.82), indicating a preference for UAA as the translation termination signal.

The observed codon usage bias is typical for angiosperm chloroplast genomes, which are generally AT‐rich and tend to use codons that match the abundant tRNA pool. This bias may enhance translation efficiency and accuracy, particularly for highly expressed genes involved in photosynthesis and other essential plastid functions. The observed bias is consistent with that reported for other *Dioscorea* species (Zhao et al. 2018; Wonok et al. 2023), further supporting the evolutionary conservation of codon usage patterns within the genus.

### 3.5 Nucleotide Diversity Analysis

To detect highly variable regions for species identification, we calculated nucleotide diversity (Pi) for 72 intergenic spacer (IGS) regions and 77 protein‐coding genes across 23 *Dioscorea* species.

The IGS regions exhibited substantially higher variation than coding regions. The most variable IGS was *rpl22*‐*rps19* (Pi = 0.11204), followed by *rps11*‐*rpl36* (0.05766), *atpA*‐*atpF_2* (0.05604), *rps2‐rpoC2* (0.05440), and *ndhJ‐ndhK* (0.05101) (Figure 6). These hypervariable IGSs, all located in the LSC or SSC regions, are promising candidates for developing DNA barcodes for species discrimination within *Dioscorea*.

**FIGURE 6:**
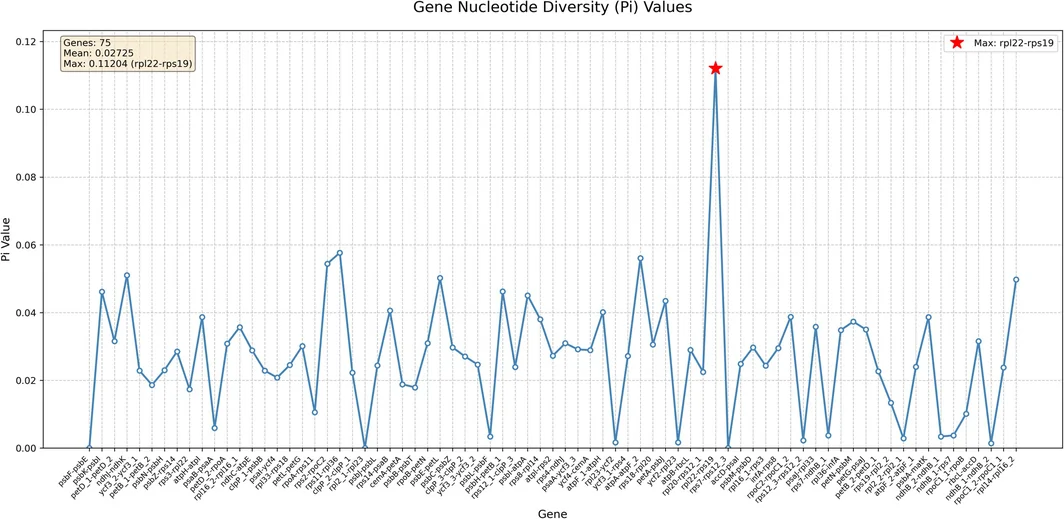
Nucleotide diversity (Pi) of intergenic spacer regions in 23 *Dioscorea* chloroplast genomes.

Among protein‐coding genes, the highest Pi values were observed for *rps19* (0.03052), *ndhF* (0.02961), *rpl20* (0.02854), and *petL* (0.02828) (Figure 7). In contrast, highly conserved genes such as *ndhB* (0.00123), *rps12* (0.00137), and *rps7* (0.00139) showed extremely low diversity, reflecting strong functional constraints. The mean Pi value of all coding genes was 0.01361. These variable coding regions, particularly *rps19* and *ndhF*, provide additional candidate markers for phylogenetic studies.

**FIGURE 7:**
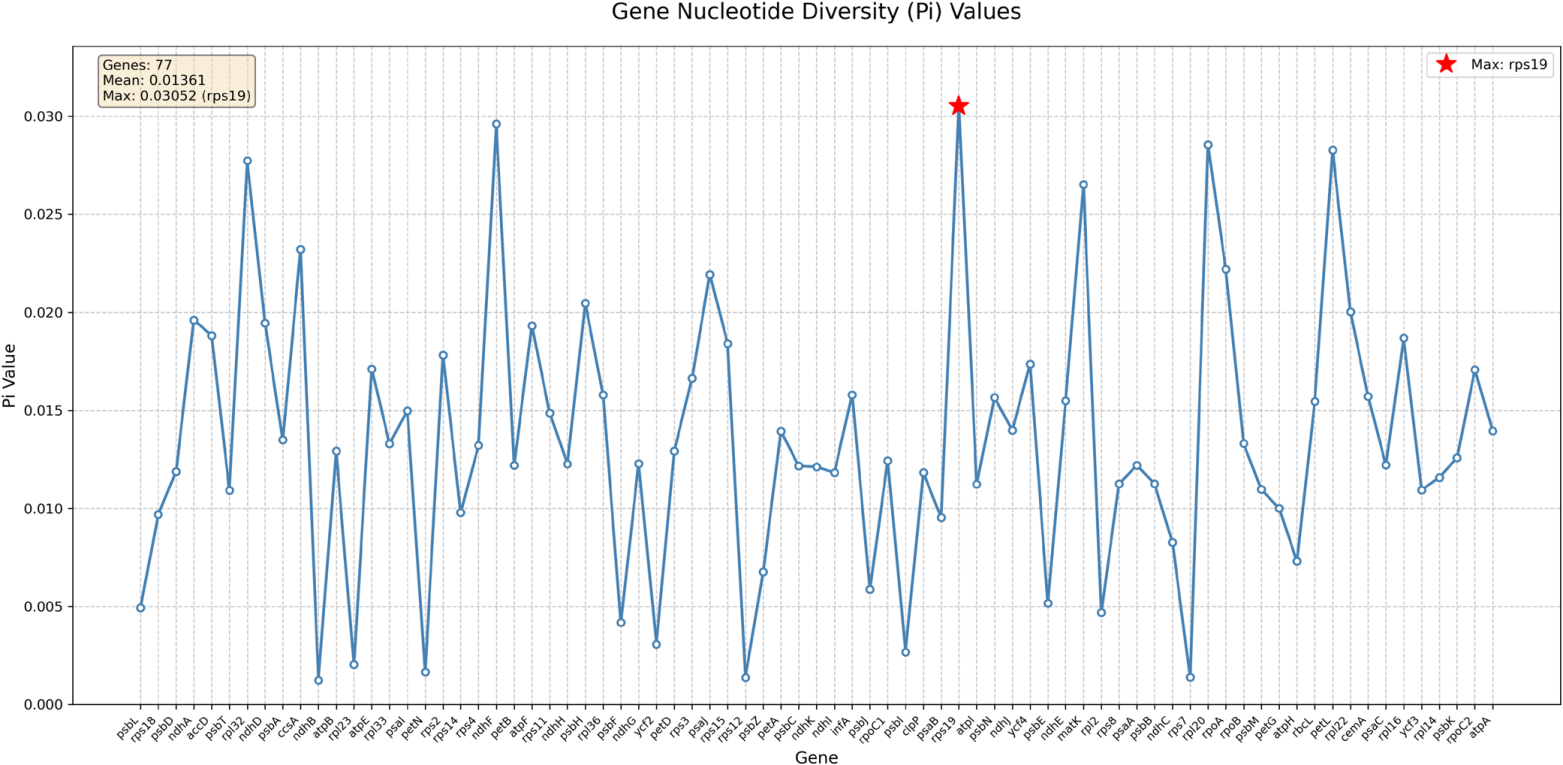
Nucleotide diversity (Pi) of protein‐coding genes in 23 *Dioscorea* chloroplast genomes. The mean Pi value (0.01361) is indicated by a dashed line.

### 3.6 Phylogenetic Tree Construction and Analysis of Species Clusters

We used maximum likelihood (ML) and Bayesian inference (BI) to reconstruct the phylogeny of 33 taxa based on complete cp genome sequences. Among these, 27 are *Dioscorea* species and six are outgroup species (three *Aletris*, two *Tacca*, and one *Trichopus*). The two methods yielded nearly identical topologies, and most nodes received high support. The ML tree is shown in Figure 8 (left), with bootstrap support (BS) values indicated at the nodes; the BI tree (right) gave posterior probabilities (PP) of 1.00 for all major clades.

**FIGURE 8:**
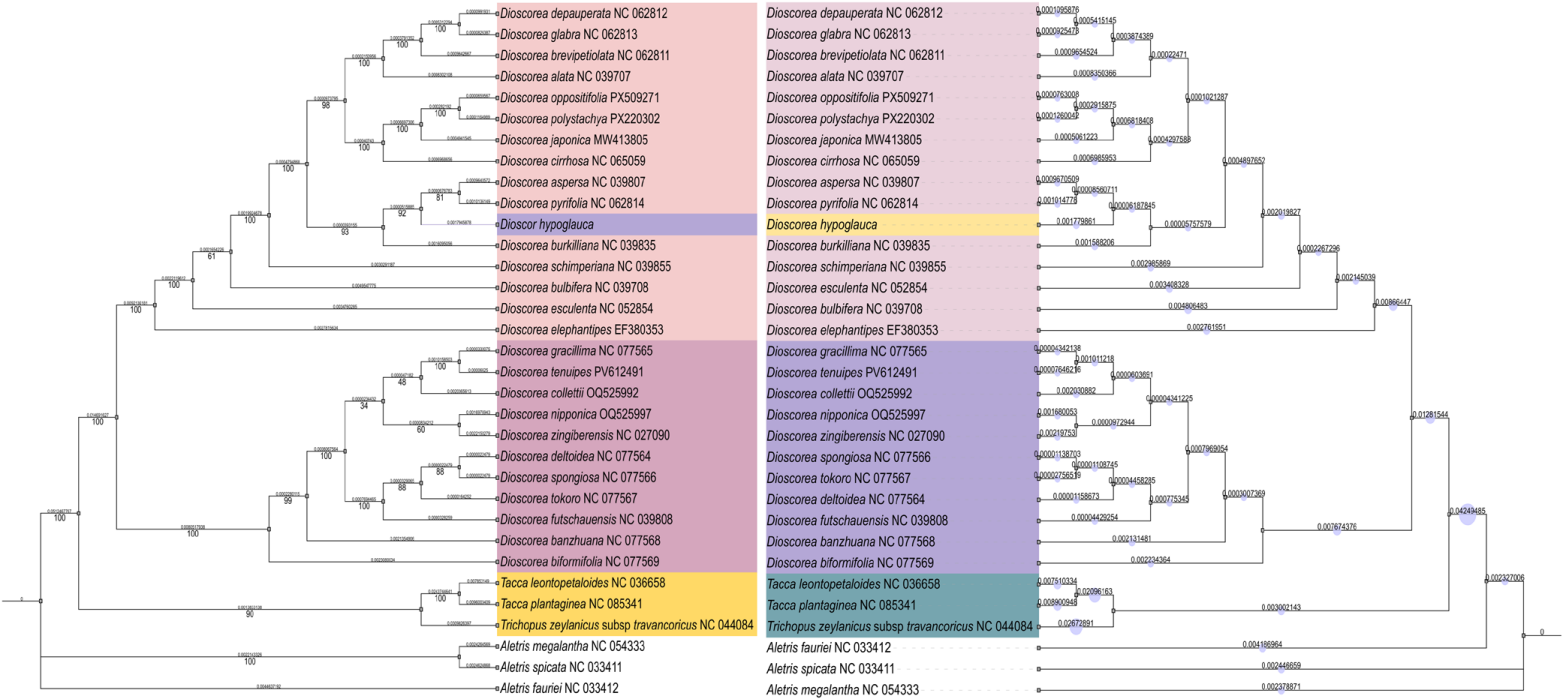
Phylogenetic trees built from complete chloroplast genome sequences of 33 taxa. The left tree comes from maximum likelihood (ML) analysis; bootstrap support values (≥ 60%) are given near each node. The right tree is from Bayesian inference (BI); posterior probabilities (≥ 0.90) appear near each node. The newly sequenced *Dioscorea hypoglauca* is shown in bold. *Aletris* species were used as outgroups.

The three *Aletris* species formed a strongly supported outgroup (BS = 100%). Within Dioscoreales, *Tacca* and *Trichopus* clustered together (BS = 100%), and this clade was sister to the monophyletic genus *Dioscorea* (BS = 100%).

The genus *Dioscorea* was resolved into two well‐supported clades. Clade I (BS = 98%) contains the majority of Asian yams, including the cultivated *D. alata* and its close relatives (*D. brevipetiolata*, *D. depauperata*, *D. glabra* ), as well as *D. cirrhosa*, *D. japonica* , *D. oppositifolia* , *D. polystachya* , and the newly sequenced *D. hypoglauca* together with *D. aspersa* , *D. pyrifolia*, and *D. burkilliana*. Within this clade, *D. hypoglauca* forms a strongly supported subclade with *D. aspersa* and *D. pyrifolia* (BS = 92%), indicating a close genetic relationship. This subclade, together with *D. burkilliana*, is sister to the *D. alata* alliance (BS = 93%).

Clade II (BS = 100%) comprises the remaining species, including the early‐diverging lineages *D. banzhuana* and *D. biformifolia* (BS = 100% and 99%, respectively), which correspond to the section Stenophora—the most basal group in Dioscorea. Within Clade II, *D. collettii*, *D. gracillima* , and *D. tenuipes* form a subclade (BS = 48%), while *D. nipponica* and *D. zingiberensis* are sister to a group containing *D. deltoidea* , *D. spongiosa* , *D. tokoro*, and *D. futschauensis* (BS = 88%–100%).

The two phylogenetic methods yielded virtually identical topologies, and all deep nodes received maximum or near‐maximum support. The robust placement of *D. hypoglauca* as a close relative of *D. aspersa* and *D. pyrifolia* clarifies its systematic position and provides a solid basis for further evolutionary and taxonomic studies of this medicinally important genus.

## 4 Discussion

The complete chloroplast genome was assembled of *Dioscorea hypoglauca*, a medicinal plant species that is endemic to southern China. The cp genome is 153,219 bp long. It has the usual quadripartite structure: an LSC of 83,692 bp, an SSC of 18,813 bp, and two IRs of 25,357 bp each. The overall GC content is 37.0%. These numbers are close to those reported for other *Dioscorea* species, for example *D. brevipetiolata* (153,485 bp, 37.0% GC) and *D. glabra* (153,503 bp, 37.0% GC) (Wonok et al. 2023). Thus, the plastome structure appears to be conserved within the genus.

We annotated 129 genes in the *D. hypoglauca* cp genome. This includes 84 protein‐coding genes, 37 tRNA genes, and eight rRNA genes. The *rps12* gene is trans‐spliced—a common situation in angiosperms (Shaw et al. 2007). Nineteen genes contain introns. Fifteen of them have one intron each; *clpP* and *ycf3* have two. Other *Dioscorea* species share a similar intron pattern, which suggests that the core photosynthetic and translational systems are under strong evolutionary constraints.

We found 35 SSRs in the *D. hypoglauca* cp genome. Mononucleotide A/T repeats are the most common type (82.86%), followed by dinucleotide (TA/AT) and trinucleotide (ATA/TAT) repeats. Many plant chloroplast genomes show a similar AT‐rich pattern (Zhao et al. 2018). One compound SSR of 82 bp (contains T‐stretches) might be a hypervariable region, potentially useful for population genetics. SSR markers derived from these loci could help distinguish *D. hypoglauca* from its adulterants in the herbal market—a strategy already applied to other medicinal plants (Li et al. 2021).

We also compared the IR boundaries across 23 *Dioscorea* species. Some features are conserved, but others are lineage‐specific. The most unusual one is the boundary shift of *ycf1* in *D. hypoglauca* : its main coding sequence shifted from the SSC region (the normal location in most species) to the IRa region, leaving only a short piece in SSC. This has not been seen in any other *Dioscorea* species examined and may represent a derived autapomorphy. Dynamic IR expansion and contraction are known to drive plastome structural evolution (Zhu et al. 2016). The *ycf1* boundary shift might have functional effects, but its adaptive significance is not yet clear and requires further investigation. At the JLB boundary, *D. hypoglauca* has a relatively contracted IRb (only 8 bp of rps19 inside IRb). In contrast, *D. zingiberensis* shows a clear IRb expansion. This suggests that such boundary shifts have happened multiple times within the genus.

Nucleotide diversity analysis across 23 species identified several hypervariable intergenic spacer regions, with *rpl22*‐*rps19* (Pi = 0.11204) being the most variable, followed by *rps11*‐*rpl36* (0.05766), *atpA‐atpF*_2 (0.05604), and *rps2‐rpoC2* (0.05440). These IGS regions are all located in the LSC or SSC. They may serve as good candidates for developing DNA barcodes to distinguish *Dioscorea* species, although their practical utility requires further validation through primer amplification tests, intra‐ versus inter‐specific divergence assessment, and barcoding gap analysis in future studies. They could also complement previously proposed markers like *ndhF* and *trnC‐ndhE* (Zhao et al. 2018; Xia et al. 2019). Among the protein‐coding genes, *rps19* (0.03052), *ndhF* (0.02961), and *rpl20* (0.02854) show the highest Pi values. This is consistent with observations in other angiosperms, where these loci tend to evolve faster (Raman et al. 2020).

Codon usage analysis revealed a strong preference for A/T‐ending codons, with 28 codons having RSCU > 1.0. For example, TTA (Leu, RSCU = 1.88), GTA (Val, 1.53), and AGA (Arg, 1.90) were highly favored, while G/C‐ending codons such as CTC (Leu, 0.40) and CGC (Arg, 0.33) were strongly avoided. This bias is typical for AT‐rich chloroplast genomes and likely reflects translational selection (Sharp and Li 1987). The preference for stop codon UAA (RSCU = 1.35) over UAG and UGA is also common among angiosperms. These data provide a foundation for predicting highly expressed genes and for optimizing transgene expression in future genetic engineering efforts.

Phylogenetic analysis using both maximum likelihood and Bayesian methods based on complete cp genomes of 33 taxa produced nearly identical topologies with high support for most nodes. The monophyly of *Dioscorea* was strongly supported (BS = 100%, PP = 1.00), and two major clades were resolved. Clade I (BS = 98%) contains *D. hypoglauca* together with *D. alata* , *D. brevipetiolata*, *D. depauperata*, *D. glabra* , *D. cirrhosa*, *D. japonica* , *D. oppositifolia* , *D. polystachya* , *D. aspersa* , *D. pyrifolia*, and *D. burkilliana*. Within this clade, *D. hypoglauca* forms a strongly supported subclade with *D. aspersa* and *D. pyrifolia* (BS = 92%, PP = 1.00), indicating a close genetic relationship. This result differs from the findings of Chen et al. (2022), who placed *D. hypoglauca* near *D. zingiberensis* based on limited markers; the discrepancy is likely due to the different molecular markers used and the expanded taxon sampling in the present study. Clade II (BS = 100%) comprises the remaining species, including the early‐diverging *D. banzhuana* and *D. biformifolia* (BS = 100% and 99%, respectively), which correspond to the section *Stenophora*—the most basal lineage of *Dioscorea* (Wilkin et al. 2005; Vinogradova et al. 2022). Overall, the robust phylogenetic placement of *D. hypoglauca* provides a solid reference for its taxonomic delimitation and for future studies on the evolution of this medicinally important genus.

In conclusion, this study provides the first complete chloroplast genome of *D. hypoglauca* , revealing its structural features, SSR composition, codon usage bias, and phylogenetic position. The hypervariable IGS regions identified here, particularly *rpl22*‐*rps19*, are promising candidates for developing DNA barcodes for species authentication. The unique boundary shift of *ycf1* adds to our understanding of plastome structural dynamics in *Dioscorea*. The genomic resources generated will facilitate the conservation, identification, and genetic improvement of this valuable medicinal plant.

## Author Contributions

**Gang Li:** conceptualization (equal), data curation (equal), formal analysis (equal), funding acquisition (equal), investigation (equal), methodology (equal), project administration (equal), resources (equal), software (equal), supervision (equal), validation (equal), visualization (equal), writing – original draft (equal), writing – review and editing (equal). **Daofeng Gong:** conceptualization (equal), data curation (equal), formal analysis (equal), investigation (equal), resources (equal), software (equal), supervision (equal), validation (equal), writing – review and editing (equal). **Xiang Cheng:** conceptualization (equal), data curation (equal), formal analysis (equal), funding acquisition (equal), investigation (equal), resources (equal), software (equal), validation (equal), visualization (equal). **Meng Li:** conceptualization (equal), data curation (equal), formal analysis (equal), funding acquisition (equal), resources (equal), software (equal), supervision (equal), validation (equal). **Na Chen:** conceptualization (equal), data curation (equal), formal analysis (equal), funding acquisition (equal), resources (equal), supervision (equal), validation (equal), visualization (equal). **Lei Cheng:** conceptualization (equal), data curation (equal), formal analysis (equal), methodology (equal), project administration (equal), resources (equal), supervision (equal), validation (equal), writing – review and editing (equal).

## Funding

This work was supported by Anhui Provincial Higher Education Quality Engineering Project (2025xqjd031) and Anhui Provincial Education Department Scientific Research Project (2025AHGXZK31358).

## Conflicts of Interest

The authors declare no conflicts of interest.

## Supporting information


**Table S1:** Species and GenBank accession numbers of the chloroplast genomes analyzed in this study.

**Table S2:** Detailed information of simple sequence repeats (SSRs) identified in the chloroplast genome of *Dioscorea hypoglauca*.

## Data Availability

The chloroplast genome sequence of *Dioscorea hypoglauca* has been submitted to NCBI (accession number: PZ427684). The Supporting Information for this article can be found online at: Tables S1 and S2.

## References

[ece374420-bib-0001] Amiryousefi, A. , J. Hyvönen , and P. Poczai . 2018. “IRscope: An Online Program to Visualize the Junction Sites of Chloroplast Genomes.” Bioinformatics 34: 3030–3031. 10.1093/bioinformatics/bty220. 29659705

[ece374420-bib-0002] Beier, S. , T. Thiel , T. Münch , U. Scholz , and M. Mascher . 2017. “MISA‐Web: A Web Server for Microsatellite Prediction.” Bioinformatics 33: 2583–2585. 10.1093/bioinformatics/btx198. PMC5870701 28398459

[ece374420-bib-0003] Caddick, L. R. , P. Wilkin , P. J. Rudall , T. A. J. Hedderson , and M. W. Chase . 2002. “Yams Reclassified: A Recircumscription of Dioscoreaceae and Dioscoreales.” Taxon 51: 103–114. 10.2307/1554967.

[ece374420-bib-0004] Chen, M. , X. Sun , J. Y. Xue , Y. Zhou , and Y. Hang . 2022. “Evolution of Reproductive Traits and Implications for Adaptation and Diversification in the Yam Genus Dioscorea L.” Diversity 14: 349. 10.3390/d14050349.

[ece374420-bib-0005] Chen, S. , Y. Zhou , Y. Chen , and J. Gu . 2018. “Fastp: An Ultra‐Fast All‐In‐One FASTQ Preprocessor.” Bioinformatics 34: i884–i890. 10.1093/bioinformatics/bty560. PMC6129281 30423086

[ece374420-bib-0006] Chinese Pharmacopoeia Commission . 2025. Pharmacopoeia of the People's Republic of China. China Medical Science Press.

[ece374420-bib-0007] Daniell, H. , S. Jin , X. G. Zhu , M. A. Gitzendanner , D. E. Soltis , and P. S. Soltis . 2021. “Green Giant‐A Tiny Chloroplast Genome With Mighty Power to Produce High‐Value Proteins: History and Phylogeny.” Plant Biotechnology Journal 19: 430–447. 10.1111/pbi.13556. PMC7955891 33484606

[ece374420-bib-0008] Daniell, H. , C. S. Lin , M. Yu , and W. J. Chang . 2016. “Chloroplast Genomes: Diversity, Evolution, and Applications in Genetic Engineering.” Genome Biology 17: 134. 10.1186/s13059-016-1004-2. PMC4918201 27339192

[ece374420-bib-0009] Frazer, K. A. , L. Pachter , A. Poliakov , E. M. Rubin , and I. Dubchak . 2004. “VISTA: Computational Tools for Comparative Genomics.” Nucleic Acids Research 32: W273–W279. 10.1093/nar/gkh458. PMC441596 15215394

[ece374420-bib-0010] Hsu, K. M. , J. L. Tsai , M. Y. Chen , H. M. Ku , and S. C. Liu . 2013. “Molecular Phylogeny of Dioscorea (Dioscoreaceae) in East and Southeast Asia.” Blumea 58: 21–27. 10.3767/000651913X673144.

[ece374420-bib-0011] Huber, H. 1998. “Dioscoreaceae.” In Flowering Plants·Monocotyledons, edited by K. Kubitzki , vol. 3, 216–235. Springer.

[ece374420-bib-0012] Jin, J. J. , W. B. Yu , J. B. Yang , et al. 2020. “GetOrganelle: A Fast and Versatile Toolkit for Accurate de Novo Assembly of Organelle Genomes.” Genome Biology 21: 241. 10.1186/s13059-020-02154-5. PMC7488116 32912315

[ece374420-bib-0013] Katoh, K. , J. Rozewicki , and K. D. Yamada . 2019. “MAFFT Online Service: Multiple Sequence Alignment, Interactive Sequence Choice and Visualization.” Briefings in Bioinformatics 20: 1160–1166. 10.1093/bib/bbx108. PMC6781576 28968734

[ece374420-bib-0014] Kumar, S. , G. Das , H. S. Shin , and J. K. Patra . 2017. “ *Dioscorea* spp. (A Wild Edible Tuber): A Study on Its Ethnopharmacological Potential and Traditional Use by the Local People of Similipal Biosphere Reserve, India.” Frontiers in Pharmacology 8: 52. 10.3389/fphar.2017.00052. PMC5306286 28261094

[ece374420-bib-0015] Li, H. , W. Xiao , T. Tong , et al. 2021. “The Specific DNA Barcodes Based on Chloroplast Genes for Species Identification of Orchidaceae Plants.” Scientific Reports 11: 1424. 10.1038/s41598-021-81087-w. PMC7809279 33446865

[ece374420-bib-0016] Lohse, M. , O. Drechsel , and R. Bock . 2007. “OrganellarGenomeDRAW (OGDRAW): A Tool for the Easy Generation of High‐Quality Custom Graphical Maps of Plastid and Mitochondrial Genomes.” Current Genetics 52: 267–274. 10.1007/s00294-007-0161-y. 17957369

[ece374420-bib-0017] Qin, Z. , Y. Du , Y. Zhong , et al. 2025. “Purification of Steroidal Saponins From Dioscoreae Hypoglaucae Rhizoma by Macroporous Resin and Evaluation of Their Anti‐Inflammatory Effect.” Journal of Chromatography B 1250: 124769. 10.1016/j.jchromb.2025.124769. 40829486

[ece374420-bib-0018] Raman, G. , K. T. Park , J. H. Kim , and S. Park . 2020. “Characteristics of the Completed Chloroplast Genome Sequence of *Xanthium spinosum* : Comparative Analyses, Identification of Mutational Hotspots and Phylogenetic Implications.” BMC Genomics 21: 855. 10.1186/s12864-020-07219-0. PMC7709266 33267775

[ece374420-bib-0019] Rambaut, A. 2014. “FigTree v1.4.4.” http://tree.bio.ed.ac.uk/software/figtree/.

[ece374420-bib-0020] Ronquist, F. , M. Teslenko , P. van der Mark , et al. 2012. “MrBayes 3.2: Efficient Bayesian Phylogenetic Inference and Model Choice Across a Large Model Space.” Systematic Biology 61: 539–542. 10.1093/sysbio/sys029. PMC3329765 22357727

[ece374420-bib-0021] Rozas, J. , A. Ferrer‐Mata , J. C. Sánchez‐DelBarrio , et al. 2017. “DnaSP 6: DNA Sequence Polymorphism Analysis of Large Data Sets.” Molecular Biology and Evolution 34: 3299–3302. 10.1093/molbev/msx248. 29029172

[ece374420-bib-0022] Sharp, P. M. , and W. H. Li . 1987. “The Codon Adaptation Index‐A Measure of Directional Synonymous Codon Usage Bias, and Its Potential Applications.” Nucleic Acids Research 15: 1281–1295. 10.1093/nar/15.3.1281. PMC340524 3547335

[ece374420-bib-0023] Shaw, J. , E. B. Lickey , E. E. Schilling , and R. L. Small . 2007. “Comparison of Whole Chloroplast Genome Sequences to Choose Noncoding Regions for Phylogenetic Studies in Angiosperms: The Tortoise and the Hare III.” American Journal of Botany 94: 275–288. 10.3732/ajb.94.3.275. 21636401

[ece374420-bib-0024] Shen, L. , J. Xu , L. Luo , et al. 2018. “Predicting the Potential Global Distribution of Diosgenin‐Contained Dioscorea Species.” Chinese Medicine 13: 58. 10.1186/s13020-018-0215-8. PMC6245757 30479655

[ece374420-bib-0025] Shi, L. , H. Chen , M. Jiang , et al. 2019. “CPGAVAS2, an Integrated Plastome Sequence Annotator and Analyzer.” Nucleic Acids Research 47, no. Webserver issue: W65–W73. 10.1093/nar/gkz345. PMC6602467 31066451

[ece374420-bib-0027] Vinogradova, G. , A. Torshilova , and E. Machs . 2022. “Flower Morphology and Phylogenetic Analysis of Some *Dioscorea* Species of the Section Stenophora (Dioscoreaceae).” Plant Systematics and Evolution 308: 42. 10.1007/s00606-022-01831-5.

[ece374420-bib-0028] Wicke, S. , G. M. Schneeweiss , C. W. dePamphilis , K. F. Müller , and D. Quandt . 2011. “The Evolution of the Plastid Chromosome in Land Plants: Gene Content, Gene Order, Gene Function.” Plant Molecular Biology 76: 273–297. 10.1007/s11103-011-9762-4. PMC3104136 21424877

[ece374420-bib-0029] Wilkin, P. , P. Schols , M. W. Chase , et al. 2005. “A Plastid Gene Phylogeny of the Yam Genus, *Dioscorea*: Roots, Fruits and Madagascar.” Systematic Botany 30: 736–749. 10.1600/036364405775097879.

[ece374420-bib-0030] Wonok, W. , R. Sudmoon , T. Tanee , S. Y. Lee , and A. Chaveerach . 2023. “Complete Chloroplast Genome of Four Thai Native *Dioscorea* Species: Structural, Comparative and Phylogenetic Analyses.” Genes 14: 703. 10.3390/genes14030703. PMC10048501 36980975

[ece374420-bib-0031] Wu, Z. Y. , and P. H. Raven , eds. 2000. Flora of China. Science Press & Missouri Botanical Garden Press.

[ece374420-bib-0032] Xia, W. , B. Zhang , D. Xing , et al. 2019. “Development of High‐Resolution DNA Barcodes for *Dioscorea* Species Discrimination and Phylogenetic Analysis.” Ecology and Evolution 9: 10843–10853. 10.1002/ece3.5607. PMC6787845 31624585

[ece374420-bib-0033] Zhao, Z. , X. Wang , Y. Yu , et al. 2018. “Complete Chloroplast Genome Sequences of *Dioscorea*: Characterization, Genomic Resources, and Phylogenetic Analyses.” PeerJ 6: e6032. 10.7717/peerj.6032. PMC6284424 30533315

[ece374420-bib-0034] Zhou, Y. , X. L. Cheng , Y. Gao , et al. 2022. “Development and Application of High‐Throughput Semi‐Automatic Genome DNA Extraction Instrument.” Journal of Plant Genetic Resources 23: 1224–1232. 10.13430/j.cnki.jpgr.20211221001.

[ece374420-bib-0035] Zhu, A. , W. Guo , S. Gupta , W. Fan , and J. P. Mower . 2016. “Evolutionary Dynamics of the Plastid Inverted Repeat: The Effects of Expansion, Contraction, and Loss on Substitution Rates.” New Phytologist 209: 1747–1756. 10.1111/nph.13743. 26574731

